# OPTN-TBK1 axis and a role for PLK1 in HSV-1 infection

**DOI:** 10.1128/mbio.02715-23

**Published:** 2023-11-29

**Authors:** Ilina Bhattacharya, Ipsita Volety, Deepak Shukla

**Affiliations:** 1 Department of Ophthalmology and Visual Sciences, College of Medicine, University of Illinois at Chicago, Chicago, Illinois, USA; 2 Department of Pathology, College of Medicine, University of Illinois at Chicago, Chicago, Illinois, USA; 3 Department of Microbiology and Immunology, College of Medicine, University of Illinois at Chicago, Chicago, Illinois, USA; Princeton University, Princeton, New Jersey, USA

**Keywords:** HSV-1, eye infection, OPTN, TBK1, PLK1, antiviral defense

## Abstract

Tank-binding kinase 1 (TBK1) plays a pivotal role as a cellular factor in regulating the immune response against herpes simplex virus type 1 (HSV-1). TBK1’s role in defending against HSV-1 is attributed to its capacity to induce the type I interferon response. Recent discoveries emphasize TBK1’s involvement in the regulation of optineurin (OPTN), an autophagy adaptor protein that plays a direct role in enhancing the antiviral response against HSV-1 through selective autophagy. Intriguingly, OPTN can also reciprocally regulate TBK1, leading to a limited understanding of the precise role of the OPTN-TBK1 axis in HSV-1 infection. In our study, we shed light on the relationship between OPTN and TBK1 in influencing the outcome of HSV-1 infection. Surprisingly, when TBK1 or OPTN was absent in cells, the spread and infectivity of HSV-1 did not align as expected. Furthermore, it was unexpected to note that cells lacking OPTN showed higher interferon expression compared to normal cells, yet they exhibited enhanced viral growth. Our investigations revealed that OPTN can directly degrade HSV-1 through autophagy even in the absence of active TBK1. Interestingly, we identified that another protein, Polo-like kinase 1 (PLK1), can compensate for the absence of TBK1 and trigger autophagy to restrict HSV-1. In summary, our findings highlight that OPTN significantly impacts the outcome of HSV-1 infection, regardless of the conventional antiviral response mediated by TBK1. Additionally, by introducing a new role for PLK1, this research provides valuable insights into the intricate interplay among OPTN, TBK1, and autophagy in shaping the course of HSV-1 infection.

**IMPORTANCE:**

Herpes simplex virus type 1 (HSV-1) is globally prevalent, with latent infections observed in up to 80% of the population. The virus is known for subverting host defense mechanisms and infiltrating the nervous system to establish latency in peripheral ganglia. Multiple stressors can reactivate the virus, and recurrent herpes has been linked to vision loss and neurodegeneration. Identifying critical host factors that limit the spread of HSV-1 and the subsequent establishment of latent infection holds the potential to drive new intervention strategies for eradicating the virus. Numerous pieces of evidence underscore the significance of Tank-binding kinase 1 (TBK1) in restricting HSV-1. Reports have also suggested that phosphorylation of optineurin (OPTN) by TBK1 is required for triggering OPTN-mediated autophagy for HSV degradation. This report adds new insights into the roles of OPTN and TBK1 in HSV-1 infection and provides proof of a TBK1-independent HSV-1 restriction through OPTN. It confirms that TBK1 activation can be substituted by PLK1 to provide protection against HSV-1. In contrast, the activation of OPTN is likely an indispensable host defense mechanism for optimal defense against HSV-1.

## INTRODUCTION

Herpes simplex virus type 1 (HSV-1) is a double-stranded DNA virus implicated in common human diseases, including fever blisters, cold sores, and cutaneous irritations (1). Additionally, it plays a prominent role as the primary etiological agent of corneal infections, consequently contributing to significant visual impairment or blindness in the developed world. Epithelial cells, including corneal epithelial cells, are the primary targets of HSV-1 infection. Moreover, HSV-1 can infect virtually all cell types *in vitro*, entering cells either through envelope fusion at the plasma membrane or a unique mode of endocytosis (2–4). Following this, the virus spreads to the peripheral nervous system, where it establishes latency in the trigeminal ganglia (1).

The induction of the interferon (IFN) pathway stands as a pivotal mechanism, orchestrating non-specific regulation of the antiviral response against incoming virions and their pathogenic effects (5, 6). For HSV-1, the recognition of its distinct molecular patterns by Toll-like receptor 3 (TLR3) and other DNA sensors triggers the activation of type I IFNs (IFN-α/β) (7–10). Within this intricate network, Tank-binding kinase 1 (TBK1) assumes a central role in directing the type I IFN response against the viral invaders (11–13). Phosphorylation of IRF3/IRF7 by TBK1 catalyzes their translocation into the nucleus, culminating in the activation of type I IFN (11–13). Studies have underscored HSV-1’s ability to subvert the IFN response by antagonizing TBK1 activity (14–17). Notably, the HSV-1 γ_1_34.5 protein serves as a suppressor of TBK1, promoting viral replication (17). Another mode of immune evasion is the inhibition of TBK1 by UL46, as demonstrated in the context of HSV-1 infection (15). Additionally, HSV-1-encoded proteins, such as Us11 and ICP27, have emerged as key players in thwarting the antiviral IFN response by targeting TBK1 (14, 16). Collectively, these investigations consistently underscore the pivotal role of TBK1 in the regulation of HSV-1 infections.

Optineurin (OPTN), an autophagy adaptor protein, functions in conjunction with TBK1(18, 19). Encoded by the OPTN gene, OPTN is a cytosolic protein (18) and a host antiviral restriction factor, impeding the replication and dissemination of HSV-1 (19). Several studies have associated OPTN with TBK1, suggesting its involvement in initiating the antiviral IFN response (20, 21). However, additional reports have highlighted OPTN’s capacity to negatively regulate the IFN response during viral infections (22, 23). TBK1 is known for inducing autophagy through the phosphorylation of OPTN (p-OPTN) (24). The OPTN-TBK1 axis has also been extensively examined for its role in autophagy activation. OPTN facilitates intracellular degradation by binding to polyubiquitinated cargoes via its ubiquitin-binding domain (18, 25, 26). TBK1’s p-OPTN at the Serine-177 site enhances OPTN’s binding affinity with polyubiquitin chains. Using a similar mechanism OPTN selectively targets essential HSV-1 proteins, gB, and VP16 and uses TBK1 as the regulating kinase for mediating the antiviral defense (19).

The intricate interplay between the IFN response and the antiviral influences modulated by these interacting proteins prompts further inquiry. Precise insights into OPTN’s interactions with TBK1 during infection, both in its presence and absence, remain elusive. Additionally, the comprehensive systemic control exerted by the OPTN-TBK1 axis over the IFN response remains to be explored. This study uncovers novel interactions between these proteins during HSV-1 infection, concluding that the absence or presence of autophagy-mediated degradation of viral proteins by OPTN significantly determines the course of HSV-1 infection, notwithstanding variations in IFN signaling. While TBK1 undoubtedly influences the antiviral IFN response during HSV-1 infection, it may not be indispensable and likely substitutable by polo-like kinase 1 (PLK1). PLK1, known for controlling cell cycle progression and regulation (27, 28), offers an alternative perspective to TBK1’s role in the HSV-1 lifecycle.

## RESULTS

### OPTN can restrict HSV-1 infection via a TBK1-independent pathway

**Fig 1:**
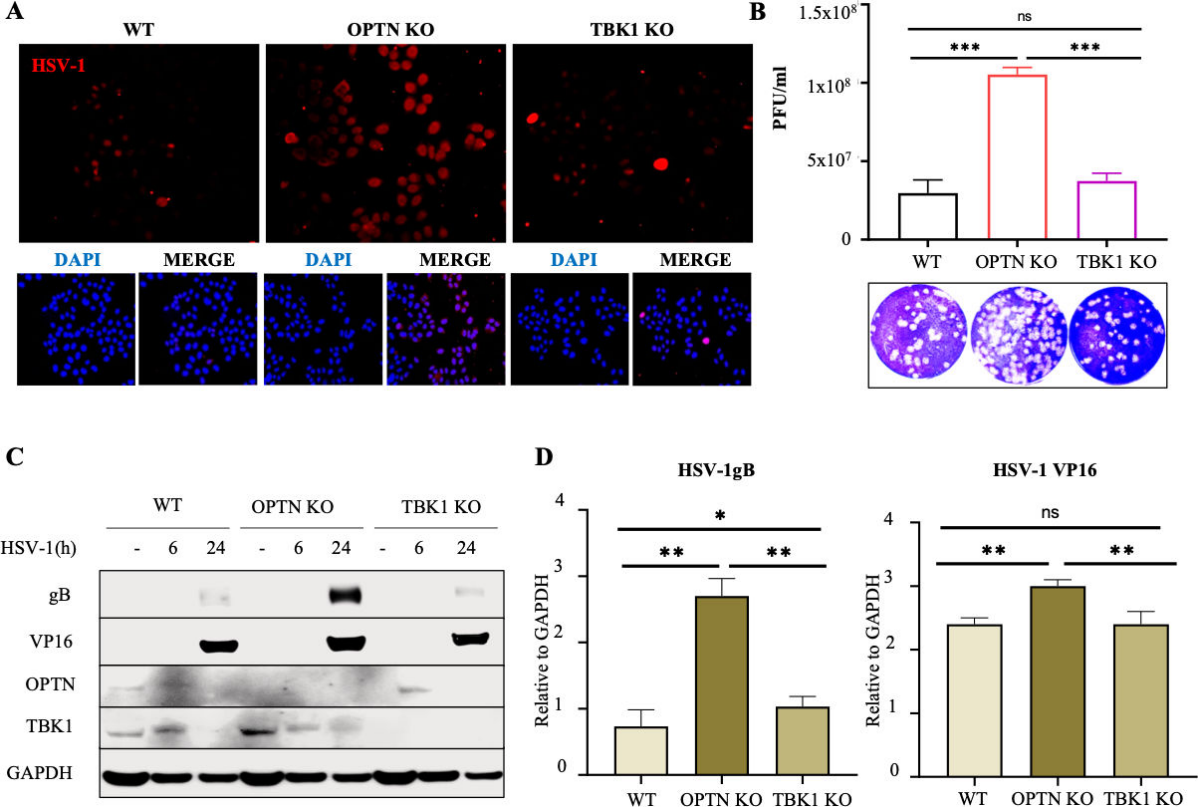
HSV-1 restriction by OPTN occurs via a TBK1-independent pathway. HeLa cells were infected with 0.1 multiplicity of infection (MOI) HSV-1 McKrae strain for 24 h. (**A**) Representative confocal imaging of WT, OPTN KO, and TBK1 KO HeLa cells. (**B**) Plaque assay data for HeLa 24 hpi. (**C**) Immunoblot for HeLa cells against HSV-1 proteins gB and VP-16. (**D and E**) Quantification of expression of gB and VP-16 relative to GAPDH from Immunoblot data using ImageJ. *n* = 3 independent replicates per group. A one-way analysis of variance test was used to determine statistical significance; *, *P* < 0.05; **, *P* < 0.01; ***, *P* < 0.001; ns not significant.

Previously, we have reported that OPTN can confer protection against HSV-1 (21). Here, to assess the role of the OPTN-TBK1 axis in the induction of this antiviral response, we resorted to the use of cells knocked out (KO) for OPTN or TBK1. Assuming equal contribution, we hypothesized a similar, higher-than-wild-type level of viral infection in both genotypes. However, the microscopy images of OPTN KO and TBK1 KO cells infected with HSV-1 revealed an unexpectedly higher spread of infection in OPTN KO cells compared to the TBK1 KO cells (Fig. 1A). The production of infectious virus particles was also increased by 140-fold in OPTN KO cells relative to TBK1 KO cells (Fig. 1B). In addition, OPTN KO cells also expressed significantly higher levels of gB and VP16, key HSV-1 viral proteins in comparison to TBK1 KO cells (Fig. 1C through E). Thus, in line with our previous report (19), we observed that OPTN KO cells promote HSV-1 spread and replication, confirming OPTN’s role as a restriction factor against HSV-1 infection. Despite the known correlation between OPTN and TBK1, we surprisingly found that viral infection in TBK1 KO cells was comparable to that in the wildtype counterparts (WT). Additionally, reduced virus spread and replication were similarly observed in TBK1 KO cells infected with other HSV-1 strains (Fig. S1). Collectively, our data raise the possibility that OPTN has the ability to restrict HSV-1 via a pathway that does not require TBK1.

### Protein knockdown in natural target cells shows limited dependence of OPTN-mediated viral restriction on TBK-1

**Fig 2:**
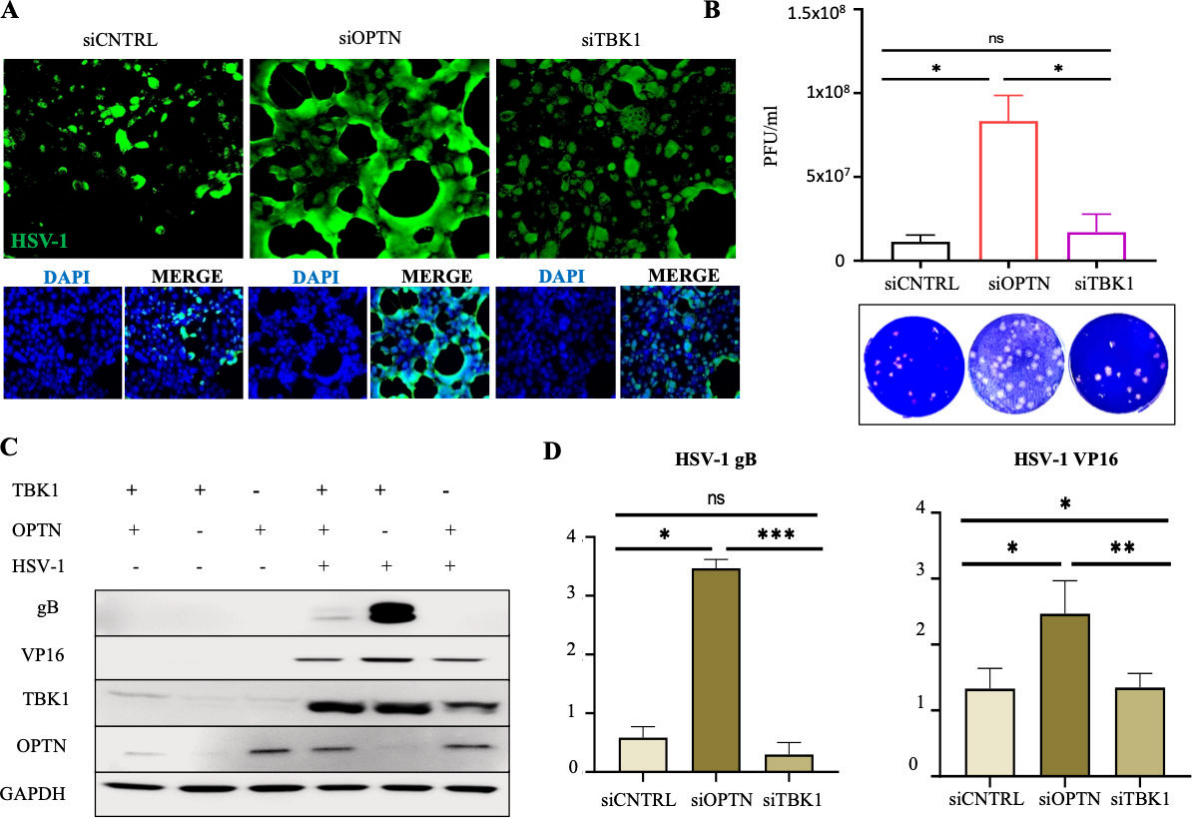
Transient knockdown of OPTN can restrict HSV-1 infection in HCE without aid from TBK1. (**A**) Representative confocal imaging of WT, siOPTN, and siTBK1 cells infected with 0.1 MOI HSV-1 McKrae for 24 h. (**B**) Plaque assay data after 24 hpi. (**C**) Immunoblot for HCE cells against HSV-1 proteins gB and VP-16. (**D and E**) Quantification of expression of gB and VP-16 relative to GAPDH from Immunoblot data using ImageJ. *n* = 3 independent replicates per group. A one-way analysis of variance test was used to determine statistical significance; *, *P* < 0.05; **, *P* < 0.01; ***, *P* < 0.001; ns not significant.

To determine whether OPTN inhibits HSV-1 infection independently of its key regulator TBK1, it is crucial to test its impact on a natural target cell line. This is particularly important due to the possibility of secondary mutations or involvement of compensatory pathways in the germline knockout models. To address this, we conducted experiments involving transient knockdown of both OPTN and TBK1 to assess their individual roles in HSV-1 restriction. Consistent with our knockout findings, our imaging experiments utilizing Human Corneal Epithelial (HCE) cells exposed to the HSV-1 McKrae strain revealed higher viral spread in siOPTN HCE cells compared to siTBK1 cells (Fig. 2A). Plaque assay data further supported these results, indicating that the knockdown of OPTN resulted in an almost 10-fold increase in production of infectious viral particles compared to siTBK1 HCE cells (Fig. 2B). Additionally, the expression levels of gB and VP16 in OPTN-deficient cells were notably higher than those in TBK1-deficient cells (Fig. 2C through E). Taken together, these results suggest that OPTN can restrict HSV-1 infection without relying on assistance from TBK1. This reinforces the notion that OPTN functions independently in inhibiting HSV-1 infection, substantiating its role as a crucial antiviral factor with or without the regulation by TBK1.

### HSV-1 proteins UL46 and γ_1_34.5 can influence cellular levels of OPTN

**Fig 3:**
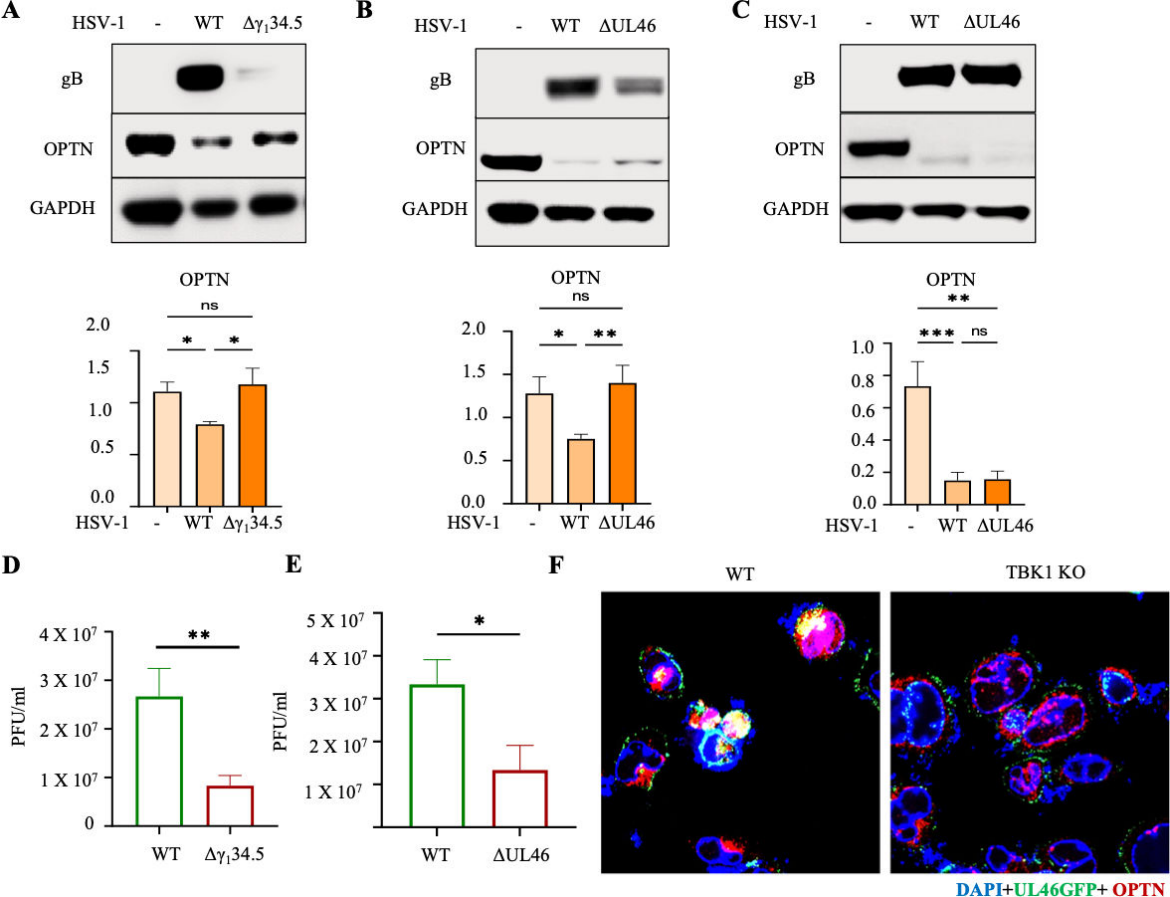
HSV-1 proteins UL-46 and γ_1_34.5 can interact with OPTN via TBK1. (**A**) Immunoblot for WT cells exposed to 0.1 MOI HSV-1 Mckrae strain (WT) and HSV-1 Mckrae Δ γ_1_34.5 strain(Δ γ_1_34.5) for 24 h. Quantification of expression of OPTN relative to GAPDH from Immunoblot data using ImageJ. (**B**) Immunoblot for WT cells was exposed to 0.1 MOI HSV-1 KOS strain (WT) and HSV-1 KOS Δ UL46 strain(ΔUL46) for 24 h. Quantification of expression of OPTN relative to GAPDH from Immunoblot data using ImageJ. (**C**) Immunoblot for TBK1 KO cells was exposed to 0.1 MOI HSV-1 KOS strain (WT) and HSV-1 KOS Δ UL46 strain (ΔUL46) for 24 h. Quantification of expression of OPTN relative to GAPDH from Immunoblot data using ImageJ; *n* = 3 independent replicates per group. A one-way analysis of variance test was used to determine statistical significance; *, *P* < 0.05; **, *P* < 0.01; ***, *P* < 0.001; ns, not significant. (**D and E**) Plaque assay data after 24 hpi. *n* = 3 independent replicates per group. A two-tailed Student’s *t* test was used to determine statistical significance; *, *P* < 0.05; **, *P* < 0.01; ***, *P* < 0.001; ns, not significant. (**F**) Representative confocal imaging of WT and TBK1 KO cells infected with HSV-1 UL46gfp.

HSV-1 proteins UL46 (15) and γ_1_34.5 (17) are known inhibitors of TBK1. To investigate their impact on OPTN, we infected cells with their respective deletion mutants (ΔUL46 and Δγ_1_34.5) and probed OPTN levels via western blotting. Interestingly, in the later stages of HSV-1 infection, we observed a significant decrease in OPTN levels. This decline is anticipated due to OPTN’s proteolytic degradation within autophagosomes, along with the specific proteins it selectively targets for removal. However, the decrease in OPTN levels was partially rescued by Δγ_1_34.5 (Fig. 3A) and ΔUL46 (Fig. 3B) infections, suggesting a potential interaction between these proteins and OPTN. Similar to a few other viral proteins, OPTN likely cargoes these proteins for selective autophagy, resulting in a corresponding reduction of OPTN levels. Elevated OPTN levels likely enhance cells’ antiviral defense, while reduced OPTN levels compromise their ability to fight infections. This is reflected by the twofold reduced infection we observed with viral mutants compared to their WT counterparts (Fig. 3D and E). Next, we aimed to determine if TBK1 activation of OPTN was necessary for this effect. To assess this, we infected TBK1 KO cells with Δγ_1_34.5 and ΔUL46 HSV-1 viral strains. Interestingly, we found no discernible differences in OPTN expression between HSV-1 parental strains and the mutant strains in TBK1 KO cells (Fig. 3C). Microscopy images of cells exposed to HSV-1 strain UL46-GFP indicated that OPTN can indeed interact with UL46 in WT cells (Fig. 3F). Conversely, limited UL46 interaction with OPTN was detected in the absence of TBK1 (Fig. 3F). This underscores that optimal targeting of HSV-1 UL46 and γ_1_34.5 by OPTN may require TBK1 activity.

### OPTN deletion or downregulation causes an aberrant IFN response

**Fig 4:**
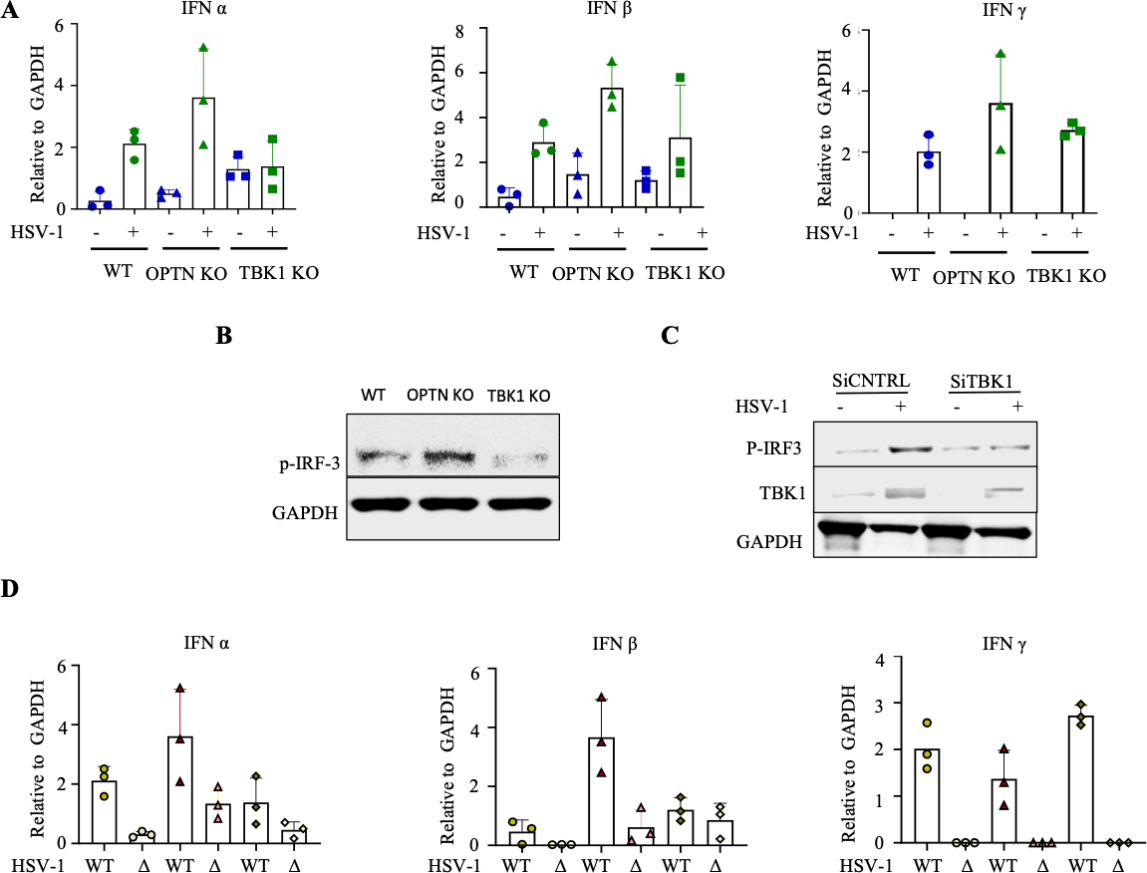
TBK1 can mediate IFN response in the absence of OPTN during HSV-1 infection. (**A**) Quantification of IFN α, IFN β, and IFN γ transcripts of cells exposed to HSV-1 infection was done by real-time PCR analysis. (**B**) Immunoblot for cells exposed to 0.1 MOI HSV-1 for 24 h. (**C**) Immunoblot for OPTN KO cells with silenced TBK1. (**D**) Quantification of IFN α, IFN β, and IFN γ transcripts of cells exposed to WT and Δ γ134.5 HSV-1 infection was done by real-time PCR analysis.

Although the infection levels differed in OPTN and TBK1 deficient cells, in this series of experiments, we aimed to evaluate whether TBK1 and OPTN work collectively to trigger an IFN response against HSV-1. Consistent with previous reports of TBK1 being a key mediator of the IFN response, we observed a decrease in IFN transcripts and p-IRF3 expression in TBK1-deficient cells (Fig. 4A and B). Interestingly, our transcript data indicated an increase in both type I and type II IFN responses in OPTN-deficient cells (Fig. 4A). We also noted an increase in the phosphorylation of IRF3, which is crucial for inducing the IFN response, in OPTN-deficient mice (Fig. 4B). Next, we hypothesized that the higher IFN response in OPTN KO cells is due to OPTN’s negative regulation of TBK1, as suggested by some previous reports (20, 23). To determine if the increased IFN response in OPTN KO cells during infection depends on TBK1 expression, we silenced TBK1 in OPTN KO cells. As anticipated, p-IRF3 expression decreased in OPTN KO cells with TBK1 knockdown (Fig. 4C). Notably, our HCE data indicated a decrease in IFN levels and p-IRF3 expression in both OPTN and TBK1 deficient cells (Fig. S2). Subsequently, we assessed IFN levels between cells exposed to the Δγ_1_34.5 HSV-1 strain. We observed that regardless of TBK1 or OPTN deficiency, IFN levels were lower in cells exposed to the Δγ_1_34.5 HSV-1 strain (Fig. 4D). Together, this data reaffirms that TBK1 is necessary for the induction of the IFN response during HSV-1 infection. The variations in IFN response in OPTN deficient cells across different cell lines suggest that OPTN deletion or downregulation leads to an aberrant IFN response, indicating its role in fine-tuning the IFN response.

### TBK1 is not essential for OPTN-mediated autophagic degradation of HSV

**Fig 5:**
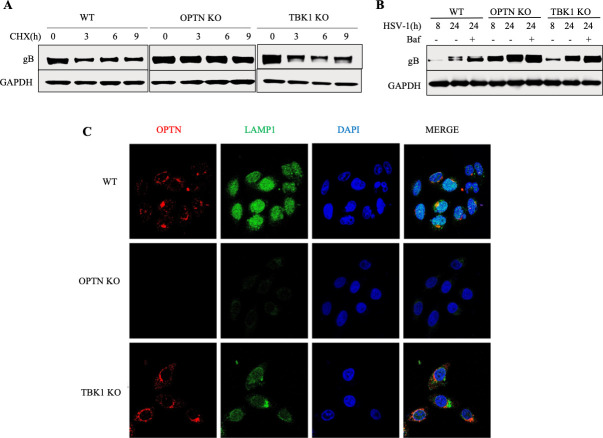
Selective autophagy of HSV-1 proteins via OPTN can occur in the absence of TBK1. WT, OPTN KO, and TBK1 KO cells were infected with 1 MOI HSV-1 infection for 12 h followed by CHX addition. (**A**) Immunoblot against HSV-1 proteins gB for cells sampled at 3 h, 6 h, and 9 h after CHX addition. (**B**) Immunoblot for WT, OPTN KO, and TBK1 KO cells was infected with 1 MOI HSV-1 infection for 8 h, followed by Bafilomycin treatment for 24 h. (**C**) Representative images from confocal imaging of WT, OPTN KO, and TBK1 KO cells exposed to 1 MOI HSV-1 for 24 h.

A previous study has linked TBK1 as a pivotal regulator of autophagy (29). To elucidate its association with the autophagic control of HSV-1 infection, we investigated the selective autophagy-mediated degradation of HSV-1 proteins in TBK1 KO cells. For this purpose, we subjected cells to HSV-1 infection, followed by the addition of cycloheximide (CHX) to impede new protein synthesis. In line with our earlier findings (19), no degradation of the HSV-1 gB protein was observed hours after the addition of CHX in OPTN KO cells (Fig. 5A). Conversely, both WT and TBK1 KO cells exhibited progressive gB degradation over time (Fig. 5A). Subsequently, we probed whether this degradation relied on autophagy. To address this question, cells were exposed to HSV-1 infection, followed by the addition of bafilomycin A1 (Baf) to obstruct the fusion between autophagosomes and lysosomes. Interestingly, the introduction of Baf in both WT and TBK1 KO cells led to the inhibition of gB degradation. This indicates that the autophagic degradation of HSV-1 protein gB can occur independently of TBK1 (Fig. 5B). Similar outcomes emerged from our imaging analysis of cells exposed to HSV-1 infection for 24 h. Here, we employed LAMP1 as a marker for autophagy activation. Notably, OPTN KO cells did not express LAMP1, aligning with our hypothesis that OPTN is essential for autophagic degradation during HSV-1 infection (Fig. 5C). Furthermore, LAMP1 expression in TBK1 KO cells was lower compared to WT cells (Fig. 5C). In conclusion, the absence of TBK1 indeed impacts the autophagy pathway. Nonetheless, our findings indicate that autophagic degradation can persist without TBK1’s involvement.

### PLK1 mediates autophagy in the absence of TBK1

**Fig 6:**
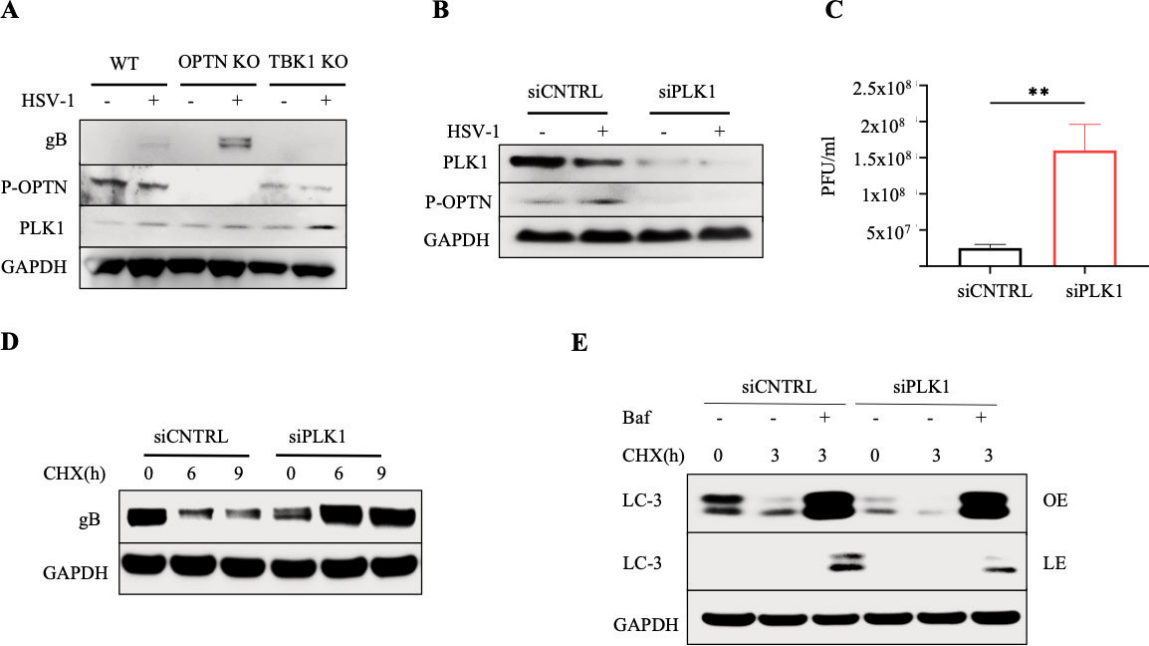
PLK1 mediates autophagy in the absence of TBK1 to facilitate HSV-1 protein degradation. (**A**) WT, OPTN KO, and TBK1 KO cells were exposed to 0.1 MOI with HSV-1, shown are the immunoblots against p-OPTN and PLK1. (**B**) SiCNTRL and siPLK1 transfected TBK1 KO cells were exposed to 0.1 MOI with HSV-1, shown are the immunoblots against p-OPTN and PLK1. (**C**) Plaque assay data for HSV-1 infected TBK1 KO cells. *n* = 3 independent replicates per group. A two-tailed Student’s *t* test was used to determine statistical significance; *, *P* < 0.05; **, *P* < 0.01; ***, *P* < 0.001; ns, not significant. (**D**) Immunoblots against HSV-1 protein gB for siCNTRL and siPLK1 transfected TBK1 KO cells were infected at 1 MOI with HSV-1 for 12 h before CHX addition to block protein synthesis, and cells were sampled at 0 h, 6 h, and 9 h after CHX addition. (**E**) Immunoblot against autophagy marker LC-3 for TBK1 KO cells infected at 1 MOI with HSV-1 for 8 h before CHX addition to block protein synthesis. In combination with CHX, either the autophagy inhibitor BafA1 or DMSO was added to cells. Cells were sampled at 8 h and 24 h after infection (LE, lower exposure; OE, overexposure).

Considering that PLK1 exhibits activities similar to TBK1, such as IFN induction (30), we hypothesized that PLK1 might serve as a candidate to induce selective autophagy of HSV-1 proteins by phosphorylating OPTN in the absence of TBK1. To investigate this, we exposed our cells to HSV-1 infection. Our immunoblot results showed the p-OPTN in TBK1 KO cells, indicating a compensatory mechanism for OPTN phosphorylation in the absence of TBK1 (Fig. 6A). Intriguingly, we also noted elevated expression of PLK1 in TBK1 KO cells during HSV-1 infection (Fig. 6A). Subsequently, we silenced PLK1 in TBK1 KO cells to assess the p-OPTN. As a result, we observed a reduction in p-OPTN expression in TBK1 KO cells with silenced PLK1 (Fig. 6B). Furthermore, plaque assays demonstrated an approximately eightfold increase in HSV-1 infection in TBK1 KO cells, where PLK1 had been silenced (Fig. 6C). To delve deeper into the impact of PLK1 silencing on autophagy, we subjected our cells to CHX treatment. Notably, the degradation of HSV-1 gB was not observed in TBK1 KO cells lacking PLK1 (Fig. 6D). Furthermore, we treated our cells with CHX followed by bafilomycin (Baf) to disrupt the fusion of autophagosomes and lysosomes. In this context, we observed diminished autophagic flux in cells with silenced PLK1 (Fig. 6E). Together, these findings suggest that PLK1-mediated p-OPTN could serve as a compensatory mechanism for TBK1’s role in activating autophagy during HSV-1 infection.

## DISCUSSION

HSV-1 shows a unique ability to circumvent the host immune system and establish latency within the trigeminal ganglion (31–33). Under the influence of stressful events and stimuli, the virus can reactivate, leading to an array of painful pathologies ranging from corneal scarring and blisters to more severe conditions like encephalitis, particularly in immunocompromised and Acyclovir-resistant patients. With approximately 80% seropositivity worldwide, the rising challenges of viral resistance, reduced drug efficacy, and limited bioavailability have cast shadows over the standard-of-care antivirals. Consequently, biomedical research must be aimed at curbing viral replication and enhancing the host’s immune response, ensuring viability, safety, and effectiveness. Notably, host proteins like OPTN and TBK1, along with signaling pathways such as the IFN response and autophagy, orchestrate robust defenses by capitalizing on intrinsic resources. The effectiveness of developed therapies hinges on these mechanisms. Unraveling these intricate mechanisms promises the development of precise strategies to counter pathogenic invasion, preventing the establishment of future latency.

Numerous studies have consistently investigated the role of TBK1 in herpes infection (13, 14, 16, 17). These studies have also shed light on TBK1’s involvement in orchestrating diverse antiviral responses, including the IFN response and autophagy (11, 12, 29). Earlier reports have additionally highlighted distinct TBK1 interacting partners, such as METTL3 and STING, and their roles in eliciting antiviral defenses against HSV-1 (14, 34, 35). In this context, we underscore the significance of another pivotal TBK1 interacting partner, OPTN, and their collaborative role in initiating antiviral immunity against HSV-1. Our prior work established the necessity of TBK1 in phosphorylating OPTN to activate autophagy, thereby curtailing HSV-1 replication and dissemination (19). Given our collected data and the cumulative insights from previous research, we anticipated observing comparable infection rates in both OPTN and TBK1 KO cells. Quite unexpectedly, our findings revealed that OPTN independently restrains HSV-1 infection, irrespective of TBK1 signaling.

In our recent study, we made a notable observation that TBK1 activity is indeed essential for triggering the type I IFN response against HSV-1. However, the degree of OPTN’s involvement in this induction seems to be contingent upon the specific cell type. Remarkably, our findings also reveal that OPTN can exert both positive and negative regulatory effects on IFN responses, influenced by the type of cell during HSV-1 infection. Moreover, our prior *in vivo* data showcases a heightened IFN response during HSV-1 infection in OPTN KO mice, underscoring the negative regulation exerted by OPTN (19). Reports have also highlighted OPTN’s role in negatively regulating IFN responses against RNA viruses (22). Collectively, it appears that OPTN’s role in mediating antiviral responses may hinge on the interplay between cell type and virus type. Further probing is imperative to discern the potential mechanism through which OPTN mediates this variable IFN response. Nonetheless, irrespective of the IFN response, a consistent pattern emerges from our observations: the lack of OPTN promotes HSV-1 infection.

Extensive literature identifies various HSV-1 viral gene products, including UL46, γ_1_34.5, Us11, and ICP27, and their well-documented role in inhibiting TBK1 activity (14–17). In our study, we set out to delve into the intriguing question of whether select HSV-1 gene products that hinder TBK1 could potentially intersect with the regulatory ambit of OPTN. Remarkably, we unveil a putative reciprocal response orchestrated by TBK1, in which it activates OPTN to interact with these viral proteins, potentially directing them toward degradation through selective autophagy.

For long, TBK1 has been hailed as central to regulating OPTN-mediated autophagy (24, 36). We conducted our study to investigate if OPTN can degrade viral proteins independent of TBK1 signaling. We demonstrate that OPTN can degrade HSV-1 proteins like gB even in the absence of TBK1. The Polo-like Kinase 1 (PLK1) is essential in mediating cell division (27, 28, 37). It has been shown to phosphorylate OPTN at the Ser-177 site during mitosis (28). In the present study, we report that both PLK1 and TBK1 phosphorylate OPTN at the same site but may have different consequences. This is interesting, given that PLK1 is considered a positive autophagy regulator (38). We hypothesized that PLK1 might mediate the autophagic degradation of HSV-1 proteins by phosphorylating OPTN even in the absence of TBK1, acting as an essential compensatory and alternate feedback pathway. We successfully established that OPTN can mediate autophagy in the absence of TBK1 with the aid of PLK1. We report PLK1’s p-OPTN occurs at the same serine site that TBK1 utilizes for HSV-1 infection. This phosphorylation event, usually responsible for mitosis, can mediate autophagic degradation of essential HSV-1 proteins and compensate for TBK1 activity in its absence.

**Fig 7:**
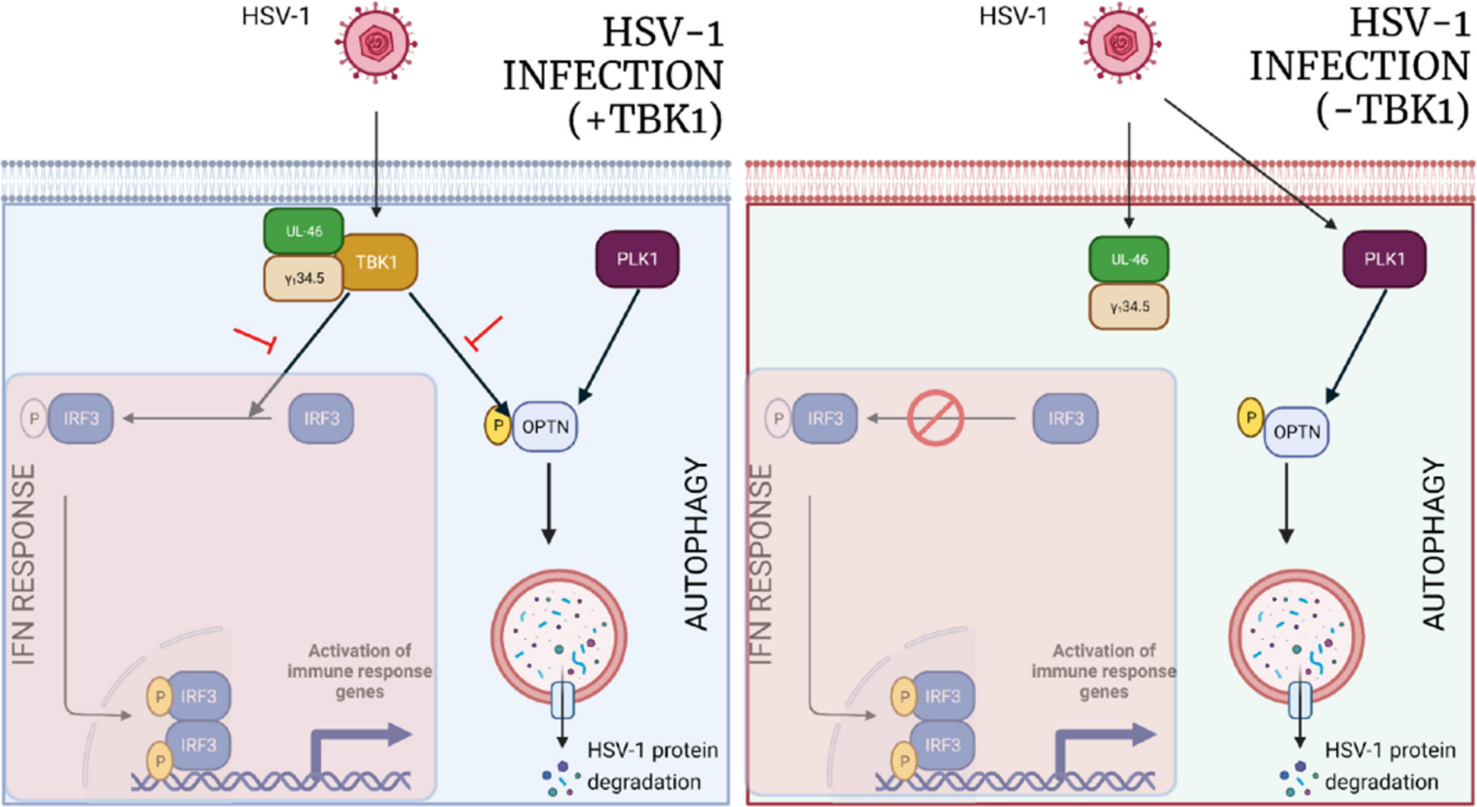
Illustration highlighting p-OPTN in the presence and absence of TBK1. In the presence of TBK1, both TBK1 and PLK1 aid in p-OPTN during HSV-1 required to trigger selective autophagy against HSV-a viral proteins. In the absence of TBK1, PLK1 can take over the TBK1 activity and phosphorylate OPTN to activate autophagy against HSV-1.

In summary, we present a new pathway of selective autophagy driven by OPTN that operates independently of TBK1, which may be redundant but sufficient in limiting HSV-1. In our model, the presence of TBK1, TBK1, and PLK1 act in concert to phosphorylate OPTN in response to HSV-1 infection. When TBK1 is absent, the onus is on PLK1 to activate protective autophagy in response to HSV-1 infection through the p-OPTN (Fig. 7). Additionally, we demonstrate that OPTN can impede HSV-1 infection regardless of the IFN response. These outcomes suggest that OPTN-facilitated autophagy might hold the answer to the progression of HSV-1 infection and the resulting disease effects. OPTN serves multiple functions, participating in various cellular pathways. The insights from this study will contribute to identifying other key contributors collaborating with OPTN to suppress infection. These insights are crucial in the development of effective pharmacological and antiviral treatments.

## MATERIALS AND METHODS

### Cells and virus strains

HeLa OPTN KO and TBK1 KO cells along with parental strain were provided by Dr. Richard Youle (National Institutes of Health). Vero cells (ATCC) were used in plaque assays and virus preparation. SV40-immortalized HCE cell lines were provided by Dr. Kozaburo Hayashi (National Eye Institute, Bethesda, MD) (39). Dr. Patricia Spear (Northwestern University) provided the HSV-1 KOS strain. The strain McKrae and McKrae γ_1_34.5-null mutant of HSV-1 was provided by Dr. Homayon Ghiasi (Cedars Sinai). Paul Kinchington (University of Pittsburgh) provided the dual tagged KOS strain of HSV-1 (RFP driven by gC promoter and GFP driven by ICP0 promoter).

The following chemicals were used in this study: Bafilomycin A1 (Sigma) to inhibit autophagic flux; CHX (Sigma) to block *de novo* protein synthesis; Lipofectamine 2000 (Thermo Fisher); and RNAiMAX (Thermo Fisher) for transfections.

### Antibodies, plasmids, and chemical reagents

The following antibodies and strains were used in this study for imaging: DAPI (D9542, Sigma) (1:1,000), mouse monoclonal to LAMP1 (Abcam, #ab25630 [H4A3]; 1:100), goat anti-mouse IgG (H+L) highly cross-adsorbed secondary antibody, Alexa Fluor 546 (Thermo Fisher, A-11030; 1:100), goat anti-rabbit IgG (H+L) highly cross-adsorbed secondary antibody, Alexa Fluor 647 (Thermo Fisher, #A-21245; 1:100), rabbit polyclonal anti-OPTN (C-terminal; Cayman Chemical, No. 100000; 1:100), and TBK1 antibody (Abclonal #A2573; 1:1,000).

The following antibodies were used for immunoblotting: mouse monoclonal anti-GAPDH (Santa Cruz, sc-69778 [7B]; 1:1,000), goat anti-mouse IgG (H+L) highly cross-adsorbed secondary antibody HRP (Thermo Fisher, 31432) (1:5,000), goat anti-rabbit IgG (H+L) cross adsorbed secondary antibody HRP (Thermo Fisher, G-21234; 1:5,000), rabbit monoclonal anti-p-S177 OPTN (Cell Signaling Technologies, 57548 S; 1:1,000), mouse monoclonal anti-HSV1 + HSV2 VP16 (Abcam, ab110226 [LP1]; 1:1,000), and rabbit polyclonal anti-OPTN (C-terminal; Cayman Chemical, No. 100000; 1:1,000).

### Western blot

Proteins were extracted from cultured cells using RIPA buffer (Sigma Aldrich; R0278-50ML). Lysis was performed with periodic vortexing on ice for 30 minutes, followed by centrifugation at 13,000 rpm for 30 minutes. Insoluble pellets were discarded, and lysates were normalized using the bicinchoninic acid (BCA) assay kit (Pierce-ThermoScientific; 23225) and then denatured at 95°C for 8 minutes using 4× lithium dodecyl sulfate (LDS) sample loading buffer (Life Technologies) and 5% beta-mercaptoethanol (Bio-Rad, Hercules, CA). Cellular proteins were separated by SDS-PAGE with NuPAGE 4%–12% Bis-Tris 1.5 mm 15-well gels (ThermoScientific) transferred to nitrocellulose using the iBlot2 system (Thermo Scientific), and membranes were blocked for 1 h in 5% milk/TBS-T, followed by incubation with primary antibody in 5% milk/TBS-T overnight at specified dilutions. Membranes were washed three times in TBS-T and then incubated with species-specific horseradish peroxidase-conjugated secondary antibodies [Jackson ImmunoResearch Peroxidase AffiniPure goat anti-mouse IgG (H+L), 115-035-146 at 1:10,000 or Peroxidase AffiniPure goat anti-rabbit IgG (H+L), 111-035-003 at 1:20,000] for 1 h at room temperature, and upon addition of SuperSignal West Femto and Pico substrates (Thermo Scientific), protein bands were detected with Image-Quant LAS 4000 biomolecular imager (GE Healthcare Life Sciences, Pittsburgh, PA).

### Quantitative polymerase chain reaction

Trizol (Thermo Scientific, 15596018) was used to extract total RNA from cultured cells, according to the manufacturer’s instructions. Complementary DNA was then produced using High-Capacity cDNA Reverse Transcription kit (Thermo Scientific, 4368814). Real-time quantitative PCR was performed with Fast SYBR Green Master Mix (Life Technologies) on QuantStudio 7 Flex system (Life Technologies).

The following human-specific primers were used in this study: IFN α forward primer 5′- AATTCTGCACCGAACTCTACC-3′; IFN α reverse primer 5′- GAAAGCGTGACCTGGTGTAT-3′; IFN β forward primer 5′-TTGAGAACCTCCTGGCTAATG-3′; IFN β reverse primer 5′- GCATCTGCTGGTTGAAGAATG-3′; IFN γ forward primer 5′- GGTCATTCAGATGTAGCGGATAA-3′; IFN γ reverse primer 5′- CACCCAAACACGAATGGAAATAG-3′; GAPDH forward primer 5′-TCCACTGGCGTCTTCACC-3′; GAPDH reverse primer 5′-GGCAGAGATGATGACCCTTTT-3′.

### Confocal immunofluorescence microscopy

Wild-type and TBK1-KO HeLa cells were cultured in 35 mm glass bottom dishes (Cellvis #D35-10-1.5-N). Cells were fixed in 4% paraformaldehyde for 10 minutes and permeabilized with 0.1% Triton-X for 10 minutes at room temperature for intracellular labeling, followed by incubation with primary antibody for 1 h at room temperature. When a secondary antibody was needed, cells were incubated with respective FITC- or Alexa Fluor 647-conjugated secondary antibody (Sigma-Aldrich F9137 or Thermo Scientific A21244) at a dilution of 1:100 for 1 h at room temperature. NucBlue Live ReadyProbes Hoechst strain (Thermo Scientific R37605) was included with secondary antibody strains when applicable, according to the manufacturer’s specifications. Samples were examined under LSM 710 confocal microscope (Zeiss) using a 63× oil immersion objective.

### Plaque assay

Viral egress was measured using the plaque assay. Monolayers of Hela WT, OPTN-KO, and TBK1-KO cells were plated in six-well plates and infected with McKrae-WT virus at MOI 0.1. Media were collected at different time points post infection and titred on Vero cells. Briefly, primary incubation of collected media was performed with Opti-MEM (Life Technologies) for 2 h. Vero cells were then incubated with growth media containing 1% methylcellulose for 72 h followed by fixing with 100% methanol and staining with crystal violet solution. Formed plaques were counted and analyzed.

### siRNA transfection

A Dicer-Substrate Short Interfering RNAs (DsiRNAs) TriFECTa Kit (IDT) with predesigned siRNA molecules was used for transfections in this study. Cells were plated and grown to 50% confluency. Cells were then transfected as per the manufacturer’s protocol using RNAiMAX at 1 µL/mL in OptiMEM (ThermoFisher). Multiple concentrations for each premade siRNA molecule were tested, and it was determined that siRNA 1 at 1 nM produced effective knockdown with minimal cell death after 48 h of transfection.

## SUPPLEMENTAL MATERIAL

Figure S1 mbio.02715-23-s0001.tiff OPTN can restrict different strains of HSV-1 without TBK1.

Figure S2 mbio.02715-23-s0002.tiff Positive regulation of IFN response in HCE cells.

ASM does not own the copyrights to Supplemental Material that may be linked to, or accessed through, an article. The authors have granted ASM a non-exclusive, world-wide license to publish the Supplemental Material files. Please contact the corresponding author directly for reuse.
