# Supplementary figures and images for: OPTN-TBK1 axis and a role for PLK1 in HSV-1 infection
Source: mBio. 2023 Nov 29;14(6):e02715-23. doi: 10.1128/mbio.02715-23 (PMC10746225; doi:10.1128/mbio.02715-23)

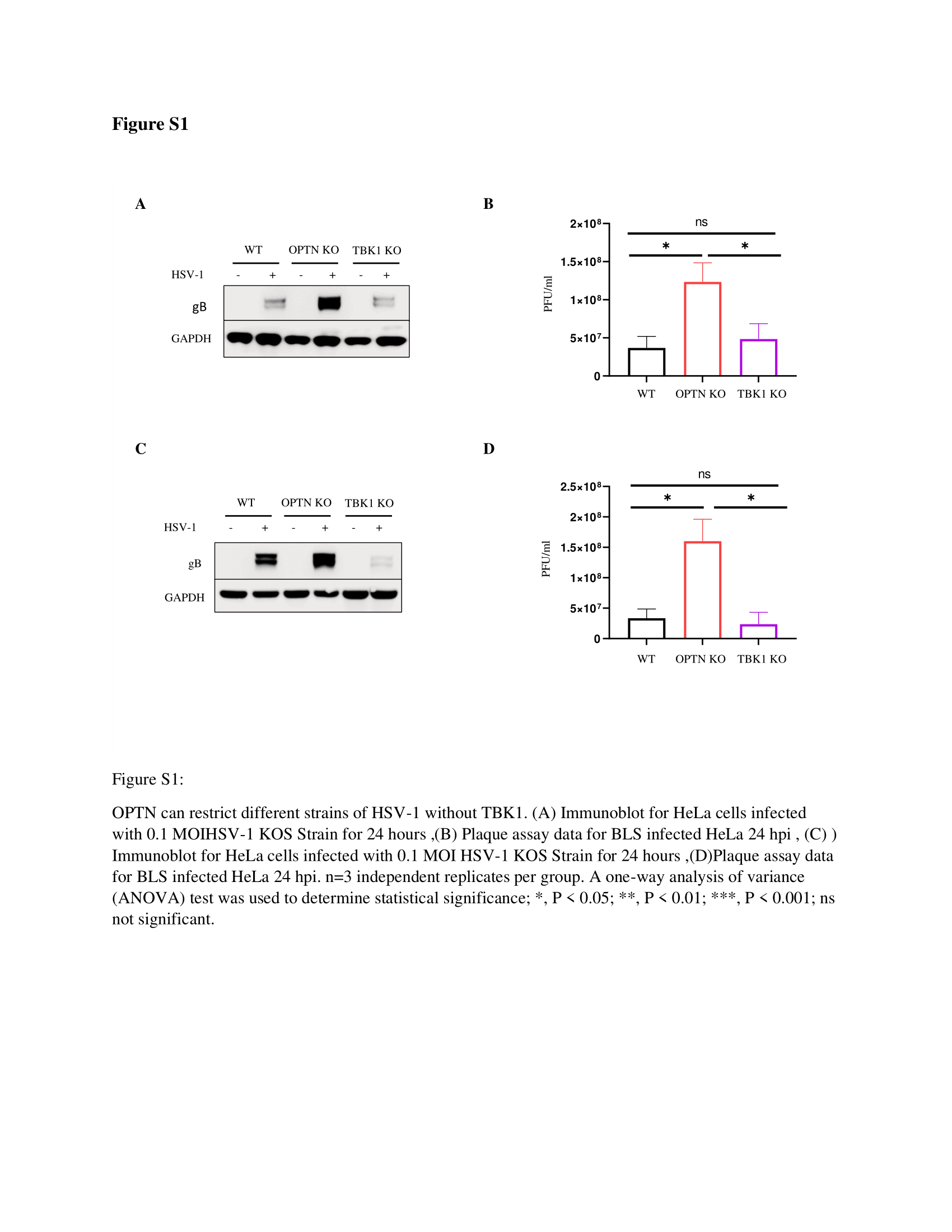

Supplement: Figure S1 — OPTN can restrict different strains of HSV-1 without TBK1. [file mbio.02715-23-s0001.tiff]

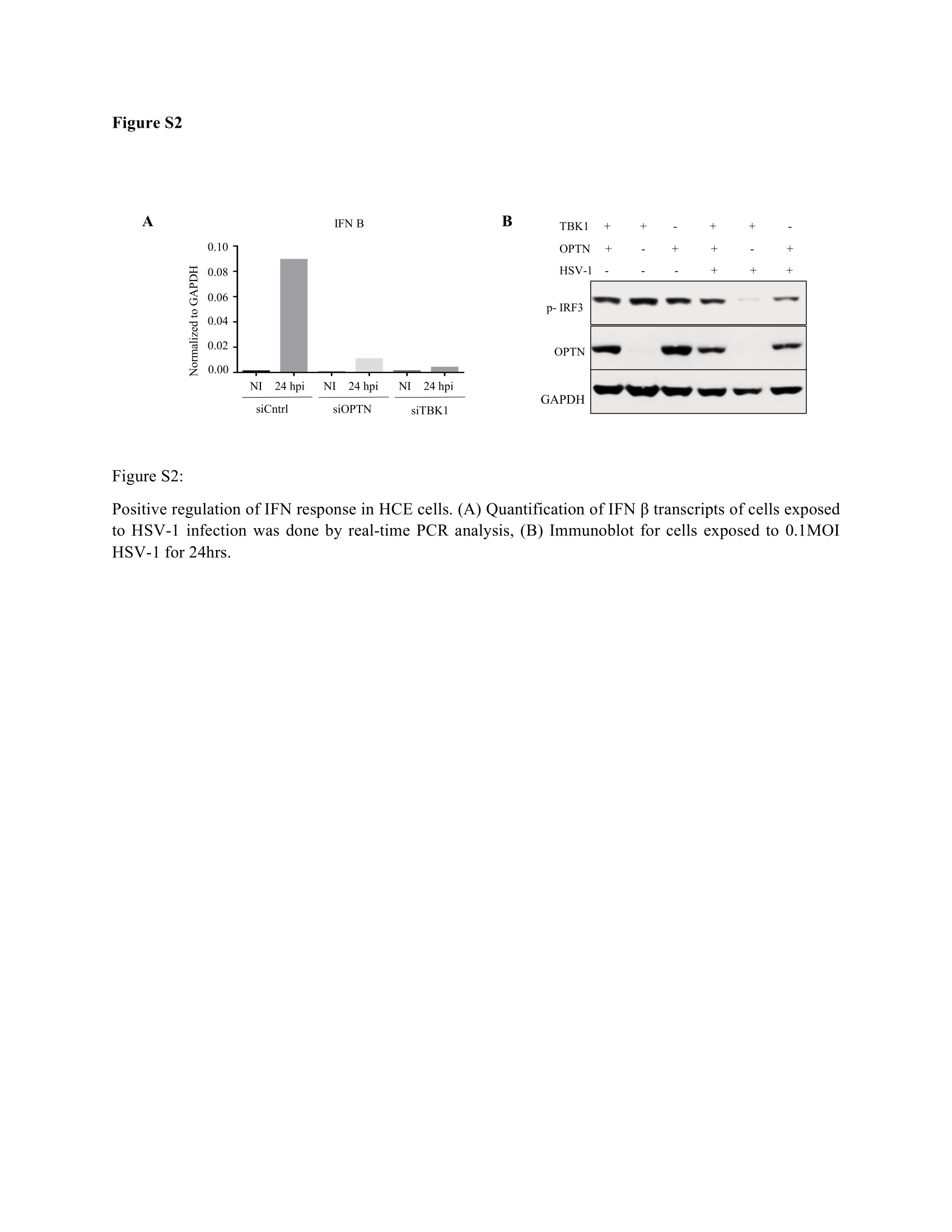

Supplement: Figure S2 — Positive regulation of IFN response in HCE cells. [file mbio.02715-23-s0002.tiff]
